# Follow-up study on health care use of patients with somatoform, anxiety and depressive disorders in primary care

**DOI:** 10.1186/1471-2296-9-5

**Published:** 2008-01-24

**Authors:** Margot WM de Waal, Ingrid A Arnold, Just AH Eekhof, Willem JJ Assendelft, Albert M van Hemert

**Affiliations:** 1 Department of Public Health and Primary Care, Leiden University Medical Center, Leiden, The Netherlands; 2 Parnassia psychomedical centre, The Hague, The Netherlands

## Abstract

**Background:**

Better management of affective and somatoform disorders may reduce consultation rates in primary care. Somatoform disorders are highly prevalent in primary care and co-morbidity with affective disorders is substantial, but it is as yet unclear which portion of the health care use may be ascribed to each disorder. Our objective was to investigate the use of primary care for undifferentiated somatoform disorders, other somatoform disorders, anxiety and depressive disorders prospectively.

**Methods:**

In eight family practices 1046 consulting patients (25–79 yrs) were screened and a stratified sample of 473 was interviewed. Somatoform disorders, anxiety and depressive disorders were diagnosed (DSM IV) using SCAN 2.1. The electronic records of 400 participants regarding somatic diseases, medication and healthcare use were available through their family physicians (FP).

**Results:**

In the follow-up year patients with psychiatric disorders had more face-to-face contacts with the FP than patients who had no psychiatric disorder: average 7–10 versus 5. The impact on the use of primary care by patients with somatoform disorders was comparable to patients with depressive or anxiety disorders. Undifferentiated somatoform disorders had an independent impact on the use of primary care after adjustment for anxiety and depressive disorders, resulting in 30% more consultations (IRR 1.3 (95% CI: 1.1–1.7)). Anxiety disorders had no independent effect.

**Conclusion:**

Health care planning should focus on the recognition and treatment of somatoform as well as affective disorders.

## Background

Psychiatric disorders may have a significant impact on consultation rates in primary care. Patients with anxiety and depressive disorders report more use of health care than patients without these disorders [1,2]. A similar assumption can be made for somatoform disorders, considering the predominant presentation of physical symptoms [3-5]. Additionally, hypochondriacal beliefs and a high somatic concern have found to be related to a high utilization of health care [6,7].

With an estimated prevalence rate between 13% and 27%, undifferentiated somatoform disorder is the most prevalent somatoform disorder in primary care [8,3]. Somatization disorder and hypochondriasis are encountered less, with prevalence rates below 5% [9]. In DSM-IV the diagnosis of undifferentiated somatoform disorder (USD) can be made when at least one medically unexplained physical symptom leads to substantial impairment for a minimum of 6 months [10]. Hence, health-seeking behaviour is not an explicit part of the definition.

The comorbidity of somatoform disorders with affective disorders is substantial [11,12]. In an earlier report on the SOUL-study, we found that in primary care one out of two patients with an anxiety/depressive disorder had a comorbid somatoform disorder [8]. It is as yet unclear which portion of the health care use may be ascribed to each disorder. High health care use in patients with an anxiety or depressive disorder might well be the result of a comorbid somatoform disorder or vice versa.

We prospectively studied the use of primary health care by patients with DSM-IV diagnoses of undifferentiated somatoform disorders, other somatoform disorders, anxiety and depressive disorders. We aimed at assessing the independent contribution of each of the disorders to primary health care utilization while controlling for somatic disease.

## Methods

### Study design

The SOmatization study of the University of Leiden (SOUL-study) was designed as a cohort study with a two-phase selection procedure [13] for clinical assessment at baseline. In the initial stage high-risk patients were identified by means of screening questionnaires. In the second phase all high-risk patients and a sample of 15% of the low risk patients were invited for a psychiatric diagnostic interview.

All interviewed patients were followed prospectively. After a follow-up of 6 months a second set of questionnaires was sent to provide for information on self-reported health care use and persistence of symptoms. After a follow-up of 12 months data were gathered on health care use in primary care using the electronic medical records of the FP.

The ethics committee of the Leiden University Medical Center approved of the study. Informed consent was obtained from the participants.

### Setting

The study took place in eight university-affiliated general practices in the vicinity of Leiden, The Netherlands, with approximately 21.500 enlisted patients.

In the Netherlands the FP is the central gatekeeper for the provision of health care. All patients are listed with one FP and primarily consult him or her for all health problems [14]. The FP indicates whether a referral to secondary care is appropriate. FPs play an important part in mental health care [15]. If a referral is indicated for a somatoform or an affective disorder, the Dutch FP has the option of a primary care mental health psychologist or a mental health service.

The electronic medical records of the FP cover all information, including reports from laboratories and specialists. As a consequence, FP records facilitate research on clinical assessment of reported symptoms.

### Patient sample

Between April 2000 and December 2001 a sample of 1778 attendees, aged 25 to 80, were sent the screening questionnaires by mail. After two weeks non-responders were sent a reminder including a copy of the questionnaires. For each practice the researchers included consecutive patients on 13 to 30 arbitrary days within a three-month period. To avoid language problems the study was limited to Dutch natives. Patients were excluded if they were unable to participate in an interview due to handicaps such as deafness, aphasia, or cognitive impairment. A total number of 1046 patients (59%) returned the questionnaire and indicated that they were willing to participate.

Participants completed the Physical Symptom Checklist (PSC) [16] and the Hospital Anxiety and Depression Scale [17] (HADS). A total score of 15 or more on the HADS or a score of 5 or more on the PSC defined the high-risk sample [18].

**Figure 1:**
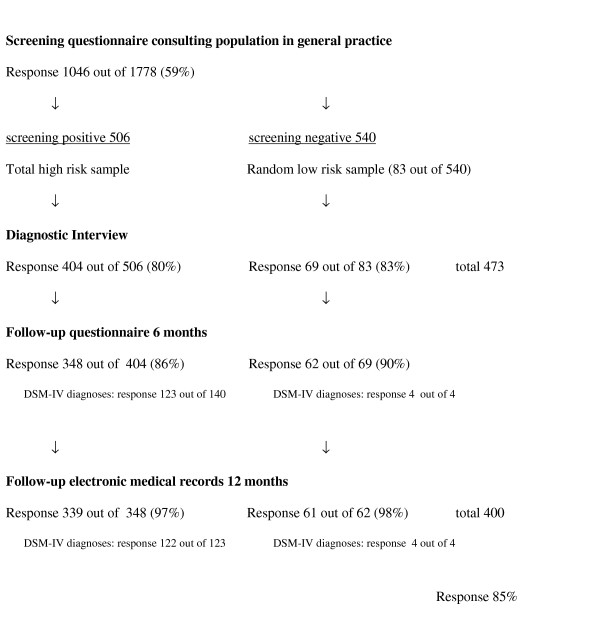
Flow-chart of the design and response of the SOUL study.

A sample of patients was contacted by mail and telephone for an interview in person at their home address and 473 out of 589 responded (80%). We obtained complete follow-up data on health care use of 400 patients (85%) (See Figure 1).

### Clinical assessment of psychiatric disorders

WHO-certified psychologists used the Schedules for Clinical Assessment in Neuropsychiatry (SCAN 2.1 [19]) for the subsequent psychiatric diagnostic interviews. Throughout the study we held regular sessions with the interviewers to maintain the diagnostic standards. During the interview patients were asked about concurrent physical illnesses, and the interviewers made the clinical decision whether symptoms were 'unexplained' or not. The FP-researcher (IAA) supervised all interviews for medical diagnostic data. Whenever necessary medical diagnostic data concerning symptoms were obtained from the individual general practitioners. When doubt remained the symptom was regarded as 'explained'.

Current disorders were diagnosed with special emphasis on impairment. Scoring algorithms on DSM-IV diagnoses were derived from the official computer program. All chronic somatoform disorders lasting at least 6 months were diagnosed: 'Acute pain disorder' and 'Somatoform disorder Not Otherwise Specified' were excluded. In this paper chronic pain disorders were regarded as undifferentiated somatoform disorders, and as such placed in the same group [20].

### Medical consumption

Self-report data on use of healthcare were obtained after a follow-up of 6 months. In the questionnaire patients were asked to report contacts with a psychiatrist, psychologist or social worker, visits to hospital or specialist and admission to hospital.

The electronic medical records of the FP were available through the central database of the family practice registration network Leiden RNUH-LEO, and provided data on medical history ('health problems'), practice contacts and prescriptions.

The FP-consultation rate was computed by counting all face-to-face contacts within office hours in the year after baseline assessment. The FP-prescription rate was computed by counting all prescriptions issued by the FP in the year after selection. The prescriptions for psycholeptics (antipsychotics, anxiolytics, hypnotics & sedatives, antidepressants) were calculated separately.

### Health status

Somatic morbidity was assessed at baseline by means of the Cumulative Illness Rating Scale (CIRS-14), a morbidity index that includes a wide range of diseases. It classifies severity of disease in terms of impairment and required treatment (ranging from 0 to 4) for 14 categories (organ systems). When two diseases are present within a category, the disease with the higher score is counted. A total morbidity score is computed by adding the severity weights [21-23]. We computerized the calculation of this score, using all available baseline data from RNUH-LEO. We excluded the category 'psychiatric' for the somatic morbidity score. The category 'psychiatric' will be presented separately.

For each patient the number of unique somatic prescriptions (issued by FP or specialist) was calculated in the year prior to selection (thus repeats were excluded). This was possible since both FPs and pharmacists use the same computer system. We assumed this number to be a measure of actual treatment of somatic morbidity, increasing the consultation rate generated by the CIRS. Moreover, new prescriptions always generate FP-contact.

### Analyses

Non-response analyses were conducted on gender, age, consultation rate and prescriptions in the year preceding baseline. Non-response on the screening questionnaire was higher among younger male patients. The consultation rate among non-responders did not differ from responders: 5.3 (se 0.2) versus 5.7 (se 0.2). Given the fact that we included consulting patients the number that had received one or more prescriptions of psycholeptics or antidepressants in the preceding year was higher than one would expect in a population sample, that is 26% and 15% respectively. Response was not affected by use of medication. Among patients with or without prescriptions of psycholeptics response rates were 61% and 58% respectively (Chisquare value 1.742, p-value = 0.19). Among patients with or without prescriptions of antidepressants response rates were 62% and 58% respectively (Chisquare value 1.086, p-value = 0.30). Non-responders to the interview scored on average 2 points higher on the distress symptom score (HADS total) than responders on the screening-questionnaire at baseline. Again, the consultation rate and use of psycholeptics or antidepressants among non-responders did not differ from responders.

Of the 473 interviewed patients 60 patients had an anxiety and/or depressive disorder and 119 patients had a somatoform disorder. The 119 patients with a somatoform disorder had a total of 121 diagnoses: 93 undifferentiated somatoform disorder, 13 chronic pain disorder, 9 hypochondriasis, 4 somatisation disorder and 2 conversion disorder (body dysmorphic disorder was not diagnosed). A more detailed description of prevalence rates and comorbidity can be found elsewhere [8]. Of the 400 interviewed patients with complete follow-up data on health care use, 20 patients had only an anxiety/depressive disorder, 94 patients had an undifferentiated somatoform disorder or chronic pain disorder of whom 27 also had an anxiety/depressive disorder, and 12 patients had other somatoform disorder of whom 4 also had an anxiety/depressive disorder. For the analyses patient numbers were weighted by the inverse of their probability of selection to adjust for differential sampling. This made figures representative for the original population.

FP-consultation rate was the main outcome measure. Because of the skewed distribution both averages and median were calculated. Incidence rate ratios (IRR) are presented to quantify the influence of the presence of psychiatric disorders on FP consultation rate. E.g., an IRR of 1.5 for disorder X is interpreted as an increase in consultation rate by 50% when disorder X is present [24]. IRRs were obtained from negative binomial regression models. Multivariate analyses were performed in STATA 9.1 by negative binomial regression models that took into account the sample scheme (survey statistics SVY: nbreg) [25]. In the first step models were built including indicator terms for the psychiatric disorders (not shown in results section). In the second step the effect of potential confounding variables was tested by including gender, age and somatic morbidity ('CIRS somatic' and 'number of unique somatic prescriptions') in the models (models 1 and 2 as reported in results section). We tested the effect of excluding the confounding variable 'number of unique somatic prescriptions', and it would only marginally change the reported results since IRRs stayed within the reported confidence intervals (not shown in results section).

## Results

Patients without psychiatric disorders had an average age of 51 years, and 23% were over 65 (Table 1). Only a small percentage of the patients with identified DSM-IV disorders were over 65: 4% of patients with anxiety or depressive disorders, 4% of patients with USD and 0% of patients with other somatoform disorders. This is in line with the low prevalence rates among the age group 65–79 years we reported earlier [8]. Somatic (co)morbidity was equally distributed over all groups. Patients with DSM-IV-defined disorders reported far more physical symptoms on the PSC. Patients with no psychiatric disorders reported an average of 4 physical symptoms, whereas those with anxiety/depressive disorder, USD and other somatoform disorders reported respectively 11, 10 and 15 physical symptoms. The latter also used more antidepressants and anxiolytics.

**Table 1:** Baseline measures. Data are weighted for the sampling scheme.

	None of the psychiatric disorders	Anxiety or depressive disorder	Somatoform disorders, undifferentiated *	Somatoform disorder, others**	Total
**Patient characteristics (questionnaire)**	(n = 274)	(n = 51)	(n = 94)	(n = 12)	(n = 400)
Gender (men)	28%	28%	20%	17%	28%
Age:					
- 65 and over	23%	4%	4%	0%	17%
- mean (se)	52 (0.8)	43 (1.3)	45 (1.0)	44 (2.8)	49 (0.6)
Physical symptoms (PSC total score)	4 (0.3)	11 (1.1)	10 (0.7)	15 (3.0)	6 (0.3)
Distress symptoms (HADS total score)	8 (0.3)	21 (1.1)	15 (0.8)	20 (2.3)	11 (0.4)

**Health status**					
Somatic morbidity:					
- CIRS somatic morbidity score	6.8 (0.2)	7.5 (0.5)	7.0 (0.4)	6.8 (0.9)	6.9 (0.2)
- Number of unique somatic prescriptions (FP or other)	5.0 (0.3)	6.3 (0.7)	6.3 (0.5)	5.7 (0.9)	5.4 (0.2)
Psychiatric morbidity:					
- CIRS psychiatric severity score	0.4 (0.0)	1.5 (0.1)	1.0 (0.1)	1.7 (0.2)	0.6 (0.0)
Use of anxiolytics (self report at time of interview)	3%	14%	15%	8%	6%
Use of antidepressants (self report at time of interview)	5%	37%	25%	42%	12%

* Undifferentiated somatoform disorders included chronic pain disorders.

** Other somatoform disorders: hypochondriasis, somatization disorder and conversion disorder.

Use of primary care during follow-up is summarized in Table 2. Patients with none of the psychiatric disorders had an average of 4.8 contacts with the FP during the follow-up year. This is in accordance with figures reported by the national statistics [26]. The patient groups with psychiatric disorders had more FP-consultations than the group without disorders. Despite the seemingly similar somatic morbidity as measured by the CIRS (Table 1), the number of non-psychiatric (somatic-related) prescriptions tended to be higher in patients with psychiatric disorders. E.g. patients with USD had an average of 12.4 (CI 95% 9.0–15.8) somatic prescriptions, while patients without psychiatric disorders had 8.0 (95% CI 6.8–9.2). Data from the follow-up questionnaire (follow-up at 6 months) showed that 39% of the patients with USD and 68% of patients with other somatoform disorders had had contact with a psychiatrist, psychologist or social worker. Of patients with anxiety or depressive disorder 59% reported mental health treatment.

**Table 2:** Follow-up measures of medical consumption. Data are weighted for the sampling scheme.

	None of the psychiatric disorder disorders	Anxiety or depressive disorder	Somatoform disorders, undifferentiated *	Somatoform disorder, others *	Total
**Medical consumption**	(n = 274)	(n = 51)	(n = 94)	(n = 12)	(n = 400)
Data from patient records (1 yr):					
Face-to-face contact with FP#:					
mean (se) median	4.8 (0.2) 4	7.5 (0.9) 7	6.9 (0.6) 5	9.8 (3.5) 5.5	5.5 (0.3) 4
Somatic prescriptions by FP^&^:					
mean (se) median	8.0 (0.6) 5	13.0 (3.6) 6	12.4 (1.7) 8	14.3 (4.2) 8.5	9.7 (0.7) 5
% use	86%	82%	95%	92%	87%
Antidepressants by FP^&^:					
mean (se) median	0.4 (0.1) 0	3.5 (1.0) 0	2.1 (0.4) 0	4.7 (2.1) 1	1.0 (0.2) 0
% use	7%	40%	32%	58%	16%
Psycholeptics by FP^&^:					
mean (se) median	0.6 (0.1) 0	4.2 (2.5) 0	2.3 (0.6) 0	4.9 (3.2) 0	1.5 (0.4) 0
% use	20%	37%	46%	25%	27%
Data from questionnaire (1/2 yr):					
- contact with psychiatrist, psychologist or social worker	14%	59%	39%	68%	23%
- visit to hospital or specialist	48%	43%	48%	33%	48%
- admission to hospital	7%	14%	10%	0%	8%

* Undifferentiated somatoform disorders included chronic pain disorders. Other somatoform disorders: hypochondriasis, somatization disorder and conversion disorder. ^# ^Consultations and visits within office hours ^&^FP prescriptions include repeats by FP of medication that was initiated by specialists

Table 3 shows the extent to which FP consultation was predicted independently by psychiatric disorders and CIRS somatic morbidity score. All models were additionally corrected for patients' age and gender and the number of unique somatic prescriptions in the year prior to follow-up (IRRs not shown).

**Table 3:** Predictors for FP-consultation rate (incidence rate ratios (IRRs) from multivariate Poisson regression models^##^).

	**FP consultation**	
	IRR	95% CI
**Model 1**^#^		
Undifferentiated somatoform disorders ^$^	* 1.3	(1.1–1.7)
Other somatoform disorders ^$$^	1.3	(0.7–2.2)
Depressive disorders	1.5	(1.0–2.3)
Anxiety disorders	0.9	(0.7–1.4)
CIRS somatic morbidity score		
- low score (<5)	1.0	reference
- intermediate score (5–8)	1.2	(1.0–1.5)
- high score (≥ 9)	** 1.6	(1.3–1.9)

**Model 2**^@^		
Psychiatric disorders		
- none of the psychiatric disorders (n = 274)	1.0	reference
- a single disorder (n = 91)	* 1.3	(1.1–1.6)
- two or more disorders (n = 35)	* 1.7	(1.2–2.5)
CIRS somatic morbidity score		
- low score (<5)	1.0	reference
- intermediate score (5–8)	1.2	0.9–1.6
- high score (≥ 9)	** 1.6	1.3–1.9

^## ^All models were additionally adjusted for the sampling scheme and for patients' age and gender and the number of unique somatic prescriptions in year prior to follow-up.

^$ ^Undifferentiated somatoform disorders included chronic pain disorders.

^$$ ^Other somatoform disorders: hypochondriasis, somatization disorder and conversion disorder.

^# ^Model 1: reference groups are patients without a disorder in the specified diagnostic group (including patients without any of the psychiatric disorders).

^@ ^Model 2: reference groups are patients without any of the specified disorders; a single disorder = a somatoform or depressive or anxiety disorder; two or more disorders = a somatoform disorder with co-morbid anxiety or depressive disorder, or anxiety disorder with co-morbid depressive disorder.

* p ≤ 0.05; ** p ≤ 0.005

In the first model (model 1) undifferentiated somatoform disorders contributed independently to the FP consultation rate: patients with an undifferentiated somatoform disorders had a 1.3 times higher consultation rate than patients without these disorders. Other somatoform disorders and depressive disorders also showed this tendency, though they were not significant. The presence of an anxiety disorder did not contribute independently to the consultation rate. Patients with a high level of somatic morbidity had a 1.6 times higher consultation rate compared to patients with a low level of somatic morbidity.

In model 2 the effect of having a single psychiatric disorder was compared to the effect of having two or more psychiatric disorders. The FP consultation rate increased 1.4 times when a single disorder (somatoform or depressive or anxiety) was diagnosed, and 1.8 times when two or more of these disorders were present.

## Discussion

### Main findings

In our sample with 1046 primary care patients, those with psychiatric disorders had more face-to-face contacts with the FP in the follow-up year than patients without psychiatric disorders. In the FP's waiting room one out of six patients will have a somatoform disorder and one out of 13 will have an anxiety or depressive disorder [8]. As we found an average consultation rate of at least 7 within a year, a FP in the Netherlands will see approximately 45 patients with these psychiatric disorders every week. Undifferentiated somatoform disorders contributed independently to a higher use of primary care after adjustment for anxiety and depressive disorders: the FP consultation rate increased by 30%.

### Limitations of the study

Despite the scale of the SOUL study, a comprehensive study including approximately 1000 consulting patients with interviews and follow-up of 400 selected patients, the power to estimate health care use for the more specific somatoform disorders such as somatization disorder was limited [8]. Disorders related to substance abuse, psychotic disorders or personality disorders were not taken into account.

For the main outcome measure, FP consultation rate, we used the reliable electronic medical records of the FP. To gather information on health care use by others than the FP we relied on self-report data. Possibly, this might have led to an over- or underestimation of this type of health care use.

When comparing consultation rates of disorders one should be aware of the effects of prescribing medication. Particularly when dealing with anxiety and depressive disorders a certain number of consultations will be initiated by the FP to monitor the prescribed medication. On the other hand, in the 'healthy group' there was a substantial number of patients with current use of psycholeptics or antidepressants, possibly disorders in remission.

### Health care use and co-morbidity

An increased use of primary health care due to depressive disorders has been reported repeatedly. In a general medical setting in the USA Luber et al. found that patients diagnosed as depressed had a significantly higher resource utilization of all types; they had an average of 5.3 visits compared to 2.9 visits for non-depressed patients [27]. Even symptoms of depression have been found to increase consultation [28,29]. Considering the high rate of co-morbidity, a substantial number of depressed patients will have an additional somatoform disorder [11,12,8].

This paper establishes that patients with undifferentiated somatoform disorders more often visited the FP than patients without psychiatric disorders, independent of the presence of co-morbid anxiety or depressive disorders. Other studies gave indications of an increased use of primary care in patients with undifferentiated somatoform disorders [4,30,3,31], but they did not take into account the considerable overlap between depressive and somatoform disorders. Overlap was taken into account in two retrospective studies. A general population study in Germany confirmed that depression and somatization both had an independent effect on health care use [32]. In primary care practices in the USA, even after adjusting for the presence of psychiatric and medical comorbidity somatizing patients had more outpatient and inpatient medical care utilization than nonsomatizing patients [33].

There are several aspects of the diagnosis 'undifferentiated somatoform disorder' that may explain the patients' reasons for consultation, such as diagnostic reassurance and management of symptoms or limitations. Rief et al. studied different aspects of illness behaviour. For somatization he found that behaviour focussed on body scanning and medication/treatment. Depression was associated with other aspects of illness behaviour that focussed on expression of symptoms and illness consequences [32]. This is in accordance to findings that somatoform disorders contribute independently to an increase in symptoms and functional limitations, after adjustment for co-morbid affective disorders [11,8].

Although mental disorders increase the utilization of general medical health care, somatization does not necessarily increase the utilization of mental health care [32,33]. In our study, 39% of patients with USD, 68% of patients with other somatoform disorders and 59% of patients with anxiety or depressive disorder received mental health treatment in primary or secondary care. In a mental health survey in the Dutch population (NEMESIS) it was found that 34% of patients with mood disorders and 18% of patients with anxiety disorders (DSM-III-R) had used ambulatory mental health care in the past 12 months, not including primary care psychologist or social worker (as we did). Moreover, they found that people who had a lifetime history of psychiatric disorder but who had been disorder-free in the past 12 months still had a four times higher use of mental health care compared to persons with no lifetime disorder [1].

## Conclusion

While we realize that the classification of somatoform disorders has been debated [34,35] and in particular alternatives of abridged forms of somatization disorder have been studied in primary care, we judged it important to diagnose somatoform disorders according to the prevailing DSM-IV classification. It was found that the prevalent entity "undifferentiated somatoform disorders" has a prognostic value in terms of health care utilization. Since both depressive and somatoform disorders contributed independently to the use of primary care, we recommend that health care planning should focus on recognition and treatment of affective as well as somatoform disorders. An integrative approach for both disorders could be advantageous for patient and doctor.

## Abbrevations

SOUL-study, SOmatization study of the University of Leiden; USD, undifferentiated somatoform disorder; FP, family physician; IRR, incidence rate ratio; HADS, Hospital Anxiety and Depression Scale; PSC, Physical Symptom Checklist; SCAN, Schedules for Clinical Assessment in Neuropsychiatry; DSM-IV, Diagnostic and Statistical Manual of Mental Disorders, 4^th ^edition; CIRS, Cumulative Illness Rating Scale.

## Competing interests

The author(s) declare that they have no competing interests.

## Authors' contributions

AMvH, IAA and MWMdW participated in the conception and design of the study, and were responsible for the acquisition and analysis of the data. All authors were involved in the analysis and interpretation of the data. MWMdW drafted the paper. All authors read and corrected draft versions of the paper and approved the final version.

## Pre-publication history

The pre-publication history for this paper can be accessed here:


